# Supplementary material for: Emergency department crowding and mortality in 14 Swedish emergency departments, a cohort study leveraging the Swedish Emergency Registry (SVAR)
Source: PLoS One. 2021 Mar 10;16(3):e0247881. doi: 10.1371/journal.pone.0247881 (PMC7946203; doi:10.1371/journal.pone.0247881)
Supplement: Attachment — Submitted filename: Response to Reviewers.docx [file pone.0247881.s001.docx]

Thank you for reviewing our manuscript, you have highlighted improvement areas that we have modified, and the manuscript has improved as a result of this! Please see our responses to your comments in blue text below.

1. Please ensure that your manuscript meets PLOS ONE's style requirements, including those for file naming. The PLOS ONE style templates can be found at

<https://journals.plos.org/plosone/s/file?id=wjVg/PLOSOne_formatting_sample_main_body.pdf> and <https://journals.plos.org/plosone/s/file?id=ba62/PLOSOne_formatting_sample_title_authors_affiliations.pdf>

The manuscript has been reformatted according to the templates except reference numbering style. We are happy to complete this as soon as the content of the manuscript has been approved by the reviewers and the text and citation ordering are final.

2. Thank you for stating the following in the Competing Interests section:

"Dr. Djärv was supported by the Stockholm County Council (clinical research appointment).

Dr. Holzmann reports receiving consultancy honoraria from Actelion, Idorsia, and Pfizer. He holds research positions funded by the Swedish Heart- Lung Foundation (grant 20170804) and the ALF agreement between the Stockholm County Council and Karolinska Institutet (grant 20170686)."

Please confirm that this does not alter your adherence to all PLOS ONE policies on sharing data and materials, by including the following statement: "This does not alter our adherence to PLOS ONE policies on sharing data and materials.” (as detailed online in our guide for authors http://journals.plos.org/plosone/s/competing-interests). If there are restrictions on sharing of data and/or materials, please state these. Please note that we cannot proceed with consideration of your article until this information has been declared.

Please include your updated Competing Interests statement in your cover letter; we will change the online submission form on your behalf.

Please know it is PLOS ONE policy for corresponding authors to declare, on behalf of all authors, all potential competing interests for the purposes of transparency. PLOS defines a competing interest as anything that interferes with, or could reasonably be perceived as interfering with, the full and objective presentation, peer review, editorial decision-making, or publication of research or non-research articles submitted to one of the journals. Competing interests can be financial or non-financial, professional, or personal. Competing interests can arise in relationship to an organization or another person. Please follow this link to our website for more details on competing interests: <http://journals.plos.org/plosone/s/competing-interests>

We have updated the Funding section in the manuscript and added that "This does not alter our adherence to PLOS ONE policies on sharing data and materials.”

3. We note that you have indicated that data from this study are available upon request. PLOS only allows data to be available upon request if there are legal or ethical restrictions on sharing data publicly. For information on unacceptable data access restrictions, please see http://journals.plos.org/plosone/s/data-availability#loc-unacceptable-data-access-restrictions.

In your revised cover letter, please address the following prompts:

a) If there are ethical or legal restrictions on sharing a de-identified data set, please explain them in detail (e.g., data contain potentially identifying or sensitive patient information) and who has imposed them (e.g., an ethics committee). Please also provide contact information for a data access committee, ethics committee, or other institutional body to which data requests may be sent.

b) If there are no restrictions, please upload the minimal anonymized data set necessary to replicate your study findings as either Supporting Information files or to a stable, public repository and provide us with the relevant URLs, DOIs, or accession numbers. Please see http://www.bmj.com/content/340/bmj.c181.long for guidelines on how to de-identify and prepare clinical data for publication. For a list of acceptable repositories, please see <http://journals.plos.org/plosone/s/data-availability#loc-recommended-repositories>.

Yes, there are ethical limitations that impact data availability. We have added a section “Data availability” explaining how to get access to the data.

**Reviewer #1:**

Thank you for a clear and well structured paper. My only difficult was trying to understand the "expected census" despite your very best efforts. I wonder if it would be helpful to give a "for instance".

Thank you! Yes, the exposure is slightly complex. We have now added an additional explanation in the “Exposure” section and in figure legends.

**Reviewer #2:**

Thank you for the opportunity to review this important and interesting study. The study is conducted in Sweden over a four year time window and uses administrative data drawn from a national quality improvement registry and linked to population vital statistics to explore the relationship between Emergency Department crowding and mortality. Drawing from over 2 400,000 visits and over 1,100,000 unique patients the study establishes definitions for the crowding exposure and using statistical methods to adjust for important covariates determines measures of association between seven day and 30 day mortality in this large robust database. Of note this is a follow-up study of a previous project conducted in Sweden where the novel crowding measure of observed over expected census was first developed though not clearly validated from the information provided in the paper. Interestingly this first report noted an association between crowding and 30 day mortality, a finding not reproduced with this report.

Thank you! The first report included all EDs in the Stockholm county so the findings in the subgroup analysis for Stockholm is probably a more relevant comparison and in line with the previous results.

The findings are somewhat mixed in that the association between crowding and mortality only achieve statistical significance in one of the four counties under consideration. This is noted in the Stockholm County where only the moderate degree of crowding was sufficiently powered to yield a robust relationship. Adding further complexity to this scenario the Stockholm County uses different criteria to code the urgency of the Emergency Department presentation. Of note this discrepancy is addressed in a sensitivity analysis which did not substantially indicate a change in the conclusions. An additional finding not well explained is how there appears to be a relationship between seven-day mortality in that county but not with the 30-day primary outcome.

In the subgroup analysis of the Stockholm county there is a significant association both with 7-day and 30-day mortality. It is in the main analysis including all counties that the results are mixed. We have now rephrased and added further clarification of the mixed results in the Discussion section.

While overall I believe this is a well conducted and nicely reported study, I do have concerns however. The primary issue I feel is the lumping of both admitted and unadmitted patients in the analysis. The reason for this is that one could argue that the impact of crowding on Emergency Department care would manifest mostly on the pressure to discharge potentially sick patients due to high hospital occupancy whereas mortality occurring for admitted patients maybe less directly linked to the quality of care provided in the Emergency Department itself and therefore the impact of crowding. Therefore I would favor a subgroup analysis based on this.

The mechanisms that may link crowding to increased mortality is still unknown but prior research show that both the pressure to discharge potentially sick patients during crowding

<https://www.bmj.com/content/342/bmj.d2983>

and inadequate care of boarded patients during crowding may contribute

<https://doi.org/10.1007/s11739-019-02231-z>

<https://doi.org/10.1111/1742-6723.13699>

We performed the subgroup analysis that you asked for and it gave the below results. We had to remove chief complaint from the model for discharged patients to get it to converge.
(We are unsure of the reason for his problem but believe that it is due to the low incidence rate for discharged patients leading to an underpowered analysis.)

**Association between crowding category and 30-day mortality (Subgroups by admission status)**

|  |  | Crowding category | | |
| --- | --- | --- | --- | --- |
| Cohort |  | 0%-75% | 75%-95% | 95%-100% |
| All hospitals | Number of deaths, n | 31,098 | 8,434 | 2,205 |
|  | Person-years at risk, n | 130,547 | 34,789 | 8,681 |
|  | Incidence rate, cases/100 person-years | 23.8 | 24.2 | 25.4 |
|  | Adjusted^1^ HR (95% CI) | Reference | 1.02 (1.00-1.05) | 1.01 (0.96-1.05) |
|  |  |  |  |  |
| Admitted | Number of deaths, n | 26,365 | 7,293 | 1,919 |
|  | Person-years at risk, n | 39,516 | 10,532 | 2,628 |
|  | Incidence rate, cases/100 person-years | 66.7 | 69.2 | 73.0 |
|  | Adjusted^1^ HR (95% CI) | Reference | 1.04 (1.01-1.07) | 1.03 (0.98-1.08) |
|  |  |  |  |  |
| Discharged | Number of deaths, n | 4,043 | 1,110 | 305 |
|  | Person-years at risk, n | 87,113 | 23,212 | 5,789 |
|  | Incidence rate, cases/100 person-years | 4.6 | 4.8 | 5.3 |
|  | Adjusted^2^ HR (95% CI) | Reference | 0.99 (0.93-1.06) | 1.00 (0.88-1.13) |

^1^ stratified by hospital, adjusted for age, sex, priority, weekend, hour, arrival mode and chief complaint

^2^ stratified by hospital, adjusted for age, sex, priority, weekend, hour, and arrival mode (chief complaint not included as model did not converge)

The higher HRs for admitted patients and lower HRs for discharged patients is in line with our expectations from our clinical experience in Sweden, where boarding is perceived to be a much bigger problem than discharging patients that should have been admitted.

However, we believe that the primary reason for the mixed results in the main analysis of this study is due to differences between the three counties. Thanks to your feedback, we performed an additional subgroup analysis by county for the admitted patients only. As a result of this we have made a revision of the manuscript. Please see the manuscript with tracked changes for details.

I also think there needs to be better justification for drawing from the most common 25 presenting complaints for this analysis. Could we not imagine a link to time sensitive conditions where treatment delays may have a greater impact on outcomes as reported in this paper? <https://pubmed.ncbi.nlm.nih.gov/31640561/>

Thank you for the article link, it seems that the study is analyzing time-sensitive ICD diagnosis groups which is an interesting approach that could be valuable in future studies!

The SVAR registry do not include information regarding ICD diagnosis but we have information regarding the presenting chief complaint in the ED. The top 25 complaints are included “with regards to the number of deaths during the study period” and not on how common the complaint was. For example, cardiac arrest is included as the number of deaths are high even if cardiac arrest is not a very common complaint. This means that we should have captured the complaints that have the largest impact on the outcome mortality.
